# Short-Term Effects of Air Pollution on Wheeze in Asthmatic Children in Fresno, California

**DOI:** 10.1289/ehp.0901292

**Published:** 2010-06-22

**Authors:** Jennifer K. Mann, John R. Balmes, Tim A. Bruckner, Kathleen M. Mortimer, Helene G. Margolis, Boriana Pratt, S. Katharine Hammond, Frederick W. Lurmann, Ira B. Tager

**Affiliations:** 1 Division of Environmental Health Sciences, School of Public Health, University of California, Berkeley, California, USA; 2 Division of Occupational and Environmental Medicine, Department of Medicine, University of California, San Francisco, California, USA; 3 Program in Public Health and Planning, Policy and Design, University of California, Irvine, California, USA; 4 Division of Epidemiology, School of Public Health, University of California, Berkeley, California, USA; 5 Division of General Medicine, Department of Internal Medicine, School of Medicine, University of California, Davis, California, USA; 6 Sonoma Technology, Inc., Petaluma, California, USA

**Keywords:** air pollution, asthma, atopy, coarse particulate matter, nitrogen dioxide, wheeze

## Abstract

**Background:**

Although studies have demonstrated that air pollution is associated with exacerbation of asthma symptoms in children with asthma, little is known about the susceptibility of subgroups, particularly those with atopy.

**Objective:**

This study was designed to evaluate our *a priori* hypothesis that identifiable subgroups of asthmatic children are more likely to wheeze with exposure to ambient air pollution.

**Methods:**

A cohort of 315 children with asthma, 6–11 years of age, was recruited for longitudinal follow-up in Fresno, California (USA). During the baseline visit, children were administered a respiratory symptom questionnaire and allergen skin-prick test. Three times a year, participants completed 14-day panels during which they answered symptom questions twice daily. Ambient air quality data from a central monitoring station were used to assign exposures to the following pollutants: particulate matter ≤ 2.5 μm in aerodynamic diameter, particulate matter between 2.5 and 10 μm in aerodynamic diameter (PM_10–2.5_), elemental carbon, nitrogen dioxide (NO_2_), nitrate, and O_3_.

**Results:**

For the group as a whole, wheeze was significantly associated with short-term exposures to NO_2_ [odds ratio (OR) = 1.10 for 8.7-ppb increase; 95% confidence interval (CI), 1.02–1.20] and PM_10–2.5_ (OR = 1.11 for 14.7-μg/m^3^ increase; 95% CI, 1.01–1.22). The association with wheeze was stronger for these two pollutants in children who were skin-test positive to cat or common fungi and in boys with mild intermittent asthma.

**Conclusion:**

A pollutant associated with traffic emissions, NO_2_, and a pollutant with bioactive constituents, PM_10–2.5_, were associated with increased risk of wheeze in asthmatic children living in Fresno, California. Children with atopy to cat or common fungi and boys with mild intermittent asthma were the subgroups for which we observed the largest associations.

Although many studies have reported that increases in ambient air pollution are associated with asthma outcomes in children, little is known about the susceptibility of subgroups of asthmatic children. In particular, the influence of allergic sensitization on the responses of asthmatic children to ambient pollutants has not been well characterized. Increased morbidity in children with asthma has been associated with particulate matter (PM) mass (Koenig et al. 2005; McConnell et al. 1999), particle number (von Klot et al. 2002), PM constituents (Delfino et al. 2003a, 2003b), nitrogen dioxide (NO_2_) (Just et al. 2002; McConnell et al. 1999), carbon monoxide (Schildcrout et al. 2006), ozone (O_3_) (Gent et al. 2003; Mortimer et al. 2000; Romieu et al. 2006), and sulfur dioxide (SO_2_) (Segala et al. 1998).

Studies that have investigated associations between the coarse fraction of outdoor PM [between 2.5 and 10 μm in aerodynamic diameter (PM_10–2.5_)] and asthma are uncommon. A review on health effects related to PM_10–2.5_ (Brunekreef and Forsberg 2005) reported only one study of asthma-related hospital admissions in children (Lin et al. 2002). Levels of PM_10–2.5_ have been associated with peripheral-blood eosinophil concentrations in adults with asthma (Yeatts et al. 2007). Increased concentrations of spores of the fungi *Cladosporium* and *Alternaria*, both of which can be found in the PM_10–2.5_ fraction, have been associated with increased asthma symptoms (Delfino et al. 1997; Ostro et al. 2001) and the occurrence of wheeze at 2 years of age (Harley et al. 2009).

Experimental studies have demonstrated that exposure to oxidant air pollutants can enhance responses of adults with allergic asthma to aeroallergens to which they are sensitized (Jenkins et al. 1999; Strand et al. 1998; Tunnicliffe et al. 1994). Epidemiologic studies have provided evidence that certain subgroups are at increased risk of asthma morbidity. For example, in a panel study of asthmatic children, personal exposure to PM ≤ 2.5 μm in aerodynamic diameter (PM_2.5_) of outdoor origin was associated with decrements in lung function, particularly in atopic boys (Delfino et al. 2004).

The Fresno Asthmatic Children’s Environment Study (FACES) was designed to investigate the effect of exposure to air pollutants on the long-term course of asthma in children. To that end, we recruited a group of children with asthma from Fresno and Clovis, California (USA), for longitudinal follow-up (Figure 1). Fresno and Clovis are located in the southern part of the San Joaquin Valley, with the Sierra Nevada Mountains to the east, surrounded by agricultural land, and situated along a major transportation corridor. As a result, the Fresno/Clovis area regularly exceeds both California and U.S. Environmental Protection Agency (EPA) ambient air quality standards for both O_3_ and PM_2.5_ [California Air Resources Board (CARB) 2009] and continues to rank as one of the most polluted areas in the country (Scorecard 2005).

A specific hypothesis of FACES is that subgroups of children with asthma are more susceptible to the effects of air pollutants. In the study we report here, we evaluated *a*) whether exposure to ambient pollution is associated with increased respiratory symptoms and *b*) whether children with atopy are more responsive to increased daily levels of air pollutants. Because PM_10–2.5_ contains bioactive constituents such as fungal spores and endotoxin, we hypothesized that such exposure would be an important contributor to symptoms. The Fresno/Clovis area offered an ideal location to study these questions because of periodic high ambient concentrations of coarse PM as well as the availability of high-quality air monitoring data.

## Methods

### Study population

A sample of 315 children with asthma was recruited in Fresno/Clovis, California, between November 2000 and April 2005. Children were recruited through school nurses, advertisements, physicians’ offices, and local media. A standardized questionnaire was used to determine eligibility. Eligibility criteria were *a*) age 6–11 years; *b*) a physician diagnosis of asthma; *c*) active asthma as indicated by current use of asthma medication, recent asthma symptoms, or asthma-related health care utilization within the previous 12 months; and *d*) current residence within a 20-km radius of the CARB air quality monitoring site in Fresno for at least 3 months. This site was also a U.S. EPA “Supersite” from 1998 to 2004. All children in the study were English speakers; parents needed to be fluent in either English or Spanish. Families who had plans to move from the area within the next year were excluded, as were children who did not spend at least 4 nights a week in one residence. To increase enrollment, siblings with asthma were included (*n* = 27) after the first year of the study. The study protocol was approved by the Committee for the Protection of Human Subjects of the University of California at Berkeley. Written informed consent for all procedures was obtained from parents/legal guardians.

### Study design

Each child and parent/legal guardian completed a baseline field office visit and was followed every 3 months during the study period. During the baseline field office visit, children had skin-prick testing (MultiTest, donated by Lincoln Labs, Decatur, IL) with 14 common local antigens (Hollister-Stier, Spokane, WA) and a histamine control. Antigens included grass, olive, rye, juniper, oak, mugwort/sagebrush, privet, cedar, house dust mite (*Dermatophagoides pteronyssinus* and *Dermatophagoides farinae*), cockroach, cat, dog, *Alternaria*, and *Cladosporium* (or *Penicillium*). Several months into the study, *Penicillium* was replaced by *Cladosporium* in the skin test panel; no children were tested with both of these antigens. At the baseline visit, participants also were trained to use an EasyOne portable spirometer (ndd Medical Technologies Inc., Zurich, Switzerland). Based on symptom frequency and severity as reported on the baseline questionnaire [see Supplemental Material, Table 1 (doi:10.1289/ehp.0901292)], we created the following asthma severity classification based on recommendations from the Global Initiative for Asthma: *a*) mild intermittent, *b*) mild persistent, and *c*) moderate or severe asthma (National Heart Lung and Blood Institute and World Health Organization 2004)

One month after the baseline visit and up to three times a year thereafter, participants completed 14-day panels of twice-daily spirometry and answered symptom questions programmed into the EasyOne spirometer with each spirometry session.

To meet the eligibility requirements for this analysis of short-term effects of air pollution on wheeze, a child needed to complete a baseline visit and at least one home visit by 31 March 2005 (last date for which air pollution data were available for this analysis). Of the 315 children in the FACES cohort, 280 (88.9%) met these requirements. Nine children with cough-variant asthma at the baseline visit who never reported wheeze as part of a panel visit were excluded from the analysis. An additional 26 children were excluded because of failure to complete any home visit before 31 March 2005.

We investigated the effect of ambient pollutant concentrations on self-reports of morning wheeze, ascertained by the question “Did you wheeze after bedtime?” programmed into the EasyOne spirometer. We limited the analysis to morning wheeze because this may identify asthmatic children who are at greater risk for adverse health outcomes (National Heart Lung and Blood Institute 1997), and evening measures are more likely to be influenced by use of rescue or controller medication during the day.

Children’s first home visits were scheduled 1 month after the baseline visit. After this visit, each child was assigned randomly to one of eight panel groups. Each panel group was scheduled for three panel periods in each of the three study seasons: winter (October–January), spring (February–May), and summer (June–September) every year.

### Ambient air pollutant measurements

Hourly, quality-assured, ambient air quality and meteorologic data collected at the Fresno “Supersite” (Watson et al. 2000) were obtained from CARB. Daily pollutant exposures were assigned to participants based on measurements at the Supersite. We used 24-hr averages for PM_2.5_ mass (micrograms per cubic meter), PM_10–2.5_ mass (micrograms per cubic meter), elemental carbon (EC; micrograms per cubic meter), NO_2_ (parts per billion), and aerosol nitrate (NO_3_; micrograms per cubic meter) and 8-hr daily maximum O_3_ (parts per billion). PM_10–2.5_ mass was determined by the difference between PM_10_ and PM_2.5_ mass measurements (Beta–Attenuation Mass Monitors; Met One Instruments, Grants Pass, OR). Black carbon (BC) was determined from aethalometer (model AE42; Magee Scientific, Berkeley, CA) measurements of the optical absorption of PM_2.5_ ambient aerosol at 880 nm. EC concentrations were estimated from BC measurements (EC = 1.19 × BC) (Chow et al. 1993). NO_3_ content in PM_2.5_ was determined from Rupprecht and Patashnick (R&P) 8400 Continuous Nitrate Analyzer (Rupprecht and Patashnick, Albany, NY) measurements adjusted for equivalency with collocated filter-based PM_2.5_ NO_3_ measurements obtained with Harvard impactors and backup filters (NO_3_ = 1.45 × R&P-measured NO_3_).

The air pollution data were subject to rigorous checks for quality assurance. These included range checks, comparison of values at nearby monitoring sites, and consistency with historical temporal and/or diurnal patterns for each pollutant.

### Statistical analysis

A multistep process was implemented to evaluate the association between the pollutants and wheeze, adjust for confounding, and identify possible effect modifiers. The steps are detailed below.

To ensure temporal sequence was preserved, a lag 0 potential exposure was considered to be the average concentration from 0800 hr the previous day to 0800 hr on the day the child answered the morning wheeze questions.

Two potential sources of confounding in these data are *a*) observed temporal covariation between wheeze occurrence and air pollutant concentrations, in part related to seasonal patterns of viral infections and outdoor allergens, and *b*) differences among the groups of children who participated on a given panel day regarding factors that may relate to risk of wheeze (e.g., age, race/ethnicity, socioeconomic status, asthma severity).

We addressed the issue of temporal covariation by the application of autoregressive, integrated, moving average (ARIMA) methods (Box et al. 1994) to daily frequency of self-reported wheeze with commercially available software (Scientific Computing Associates, Oak Brook, IL).

**Figure 1:**
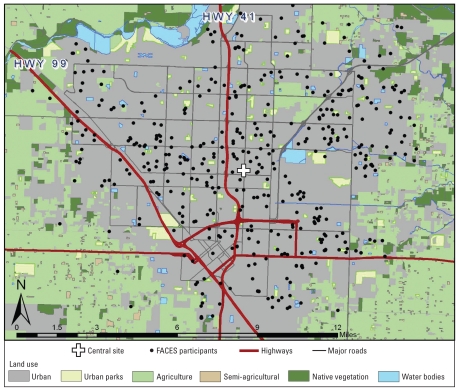
Map of study area.

The proportion of subjects who wheezed was computed for each day of the study. The ARIMA procedure requires a complete time series. We imputed values for mean daily wheeze on 121 (7.6% of 1,583) days for which no wheeze data were available with forecasting methods based on the ARIMA procedure. Once the series was complete, we performed the ARIMA procedure. Next, we examined the residual values for the proportion of subjects who wheezed on a given day (i.e., difference between the ARIMA-fitted and the observed data) to ensure that they exhibited no autocorrelation [see Supplemental Material, Figure 1 (doi:10.1289/ehp.0901292)]. We then used the ARIMA-fitted values of mean daily frequency of wheeze as an independent variable in the analyses of pollutant associations with wheeze.

The following groups of candidate covariates were considered, to adjust for differences among the children: demographic variables (home ownership, income level, race/ethnicity), personal characteristics [age at the time of the home visit, health history, asthma severity at baseline, age at asthma diagnosis, eczema, rhinitis, medication use (beta-agonists, inhaled steroids, and other asthma medications, including leukotriene blockers, were examined separately), maternal and paternal history of asthma], and home characteristics [smoking in the home, presence of pets (cat, dog, bird, rodent)]. Two types of possible empirical confounders were also considered: dummy variables for the home visit group to which each child was assigned randomly, and the 6-month interval of entry into the cohort from November 2000 through 31 March 2005 [for additional description of ARIMA methodology, see Supplemental Material (doi:10.1289/ehp.0901292)].

All analyses other than the ARIMA procedure were conducted with SAS (version 9.1; SAS Institute, Inc., Cary, NC). To be considered for model inclusion, each covariate was regressed against wheeze. Any covariate that had a *t*-statistic associated with a *p*-value of < 0.10 was retained for the next stage of model selection, a stepwise procedure with backward selection; the fitted term for wheeze was forced into the models. The final model included the fitted term for wheeze; 1-hr minimum temperature; terms for white race, sex, and moderate or severe asthma severity at baseline; a dummy variable for one of the panel groups; and a dummy variable for one of the 6-month cohorts. To account for repeated measures, the covariates and pollutant variables were then evaluated with SAS using PROC GENMOD (SAS Institute Inc.) with the logit link and the empirical option. With this approach, standard errors do not depend on correct specification of the variance-covariance matrix describing the correlation structure among the repeated measures. The inferences reported are based on an independent correlation structure for the variance-covariance matrix. A significance level of α = 0.05 was used.

To evaluate the association between each single pollutant and wheeze, each regression contained a single pollutant term with a lagged (0–14 days) or moving-average (2–14 days) value. To ensure that the associations were based on changes in air pollution experienced by the participants during the study period, the odds ratios (ORs) were computed for the 90th percentile of daily differences for the lag/moving average of each pollutant. To have a fixed point of comparison for associations for each subgroup, we identified lags associated with the largest coefficients within the first 7 days in the full cohort analysis (“representative lag”). Two-pollutant models were considered when single-pollutant effects were statistically significant.

We tested for effect modification in the following subgroups: *a*) children who were skin-test positive to at least one of the 14 antigens included in the skin-test panel, *b*) children who were skin-test positive to specific antigens, *c*) children with a history of allergic rhinitis or eczema, and *d*) children who had either mild intermittent or moderate-severe asthma at baseline. Differences in the strength of the associations between pollutant exposures and frequency of wheeze were also investigated across sex, race/ethnicity, income, and pet ownership.

## Results

The total number of calendar days with wheeze data during the study period was 1,462, resulting in 15,252 child-panel days. The participating children completed a mean of 5.2 panels (ranging from 1 to 12) during the study period. The average number of days of wheeze per panel was 1.6 (ranging from 0 to 14). Table 1 lists the characteristics of the participants. Eligible children (mean ± SD, 8.1 ± 1.7 years of age) were largely Hispanic (40.0%) and non-Hispanic white (41.1%); 20% were from families with annual incomes < $15,000. At baseline, 28.2% were classified as having mild, intermittent asthma. Almost two-thirds (63.3%) of the cohort was sensitized to one or more antigens [see Supplemental Material, Table 2 (doi:10.1289/ehp.0901292)]. Most of the children had a current prescription for inhaled steroids (74.3%), and more than one-third of participants had used oral prednisone in the 12 months before the baseline interview (38.0%).

Time series of NO_2_, PM_2.5_, NO_3_, and EC daily concentrations exhibited peaks during the cold season (October–February), whereas O_3_ and PM_10–2.5_ concentrations were highest in the warm season (April–October) [see Supplemental Material, Figure 2 (doi:10.1289/ehp.0901292)]. During the cold season, the mean daily concentration of PM_2.5_ was above the current National Ambient Air Quality Standard (NAAQS) 24-hr standard of 35 μg/m^3^ (mean ± SD, 37.3 ± 24.4 μg/m^3^). Similarly, in the warm season, nearly 32% of O_3_ concentrations were at or above the current NAAQS 8-hr standard of 75 ppb. Conversely, all the NO_2_ values were well below the 53 ppb current NAAQS annual standard over the entire time period. Table 2 displays the distribution of pollutant concentrations during the full year. NO_2_ was moderately correlated with PM_2.5_, NO_3_, and EC. The pollutants that peak in the cold season (NO_2_, PM_2.5_, NO_3_, and EC) were weakly correlated with PM_10–2.5_ and were inversely and moderately correlated (−0.56 to −0.31) with O_3_ (see Supplemental Material, Table 3).

There was more variability in mean daily wheeze in the first year of the study, presumably because there were fewer panel visits scheduled and fewer participants in the study during that year. The filtered wheeze time series after the ARIMA did not exhibit autocorrelation [see Supplemental Material, Figure 1 (doi:10.1289/ehp.0901292)].

NO_2_ lags from 1 to 7 days (except for the 4-day lag) and moving averages from 3 to 12 days were associated with increased odds of wheeze (*p* < 0.10), with the largest OR observed for the 2-day lag [OR = 1.10 per 90th percentile of absolute differences in daily concentrations (8.7 ppb), 95% confidence interval (CI), 1.02–1.20]. Three- to 12-day moving average coefficients of NO_2_ were 50–400% greater than individual-day lag coefficients and steadily increased in magnitude as the size of the moving average increased; however, the ORs for moving averages were much smaller, given the smaller absolute increment represented by a 1-day increase in the longer moving averages. PM_10–2.5_ was associated significantly with wheeze for 3- to 5-day lags, with the largest OR at 3 days [OR = 1.11 per 90th percentile of absolute differences in daily concentrations (14.7 μg/m^3^); 95% CI, 1.01–1.22]. None of the moving averages for PM_10–2.5_ was statistically significant. We observed no statistically significant associations with wheeze for NO_3_, EC, PM_2.5_, and O_3_ for the group as a whole, although the ORs for NO_3_, EC, and PM_2.5_ were similar to those for PM_10–2.5_ (Table 3) [for complete results, see Supplemental Material, Table 5 (doi:10.1289/ehp.0901292)].

Three subgroup analyses showed evidence of effect modification. Children who were skin-test positive to cat (*n* = 49) and *Alternaria* or *Cladosporium* (*n* = 85) were more likely to wheeze with increasing pollutant concentrations than those who were skin-test negative to these antigens, consistent with our *a priori* hypothesis. Atopy in general (classified as skin-test positive to any of the 14 antigens) was not an effect modifier, nor was skin-test positivity to any pollen or self-reported history of allergic rhinitis or eczema. Children who had mild intermittent asthma at baseline and boys also were more likely to wheeze than were children with more severe asthma or girls, respectively. Based on these results, we identified the group of boys with mild intermittent asthma (*n* = 47) as another susceptible group. Table 4 presents the ORs and 95% CIs for the “representative lag” (associated with the largest statistically significant OR) for the three subgroups that showed evidence of effect modification. Exposure to five of six pollutants was associated significantly with wheeze in at least one of these subgroups for a wide range of lags and moving averages [see Supplemental Material, Tables 6–8 (doi:10.1289/ehp.0901292)].

Relative to the entire study group results, odds of wheeze were 15–20% greater and statistically significant for EC, NO_2_, NO_3_, and PM_10–2.5_ when the analysis was restricted to children allergic to cat. Children allergic to *Alternaria* or *Cladosporium* were more likely than nonallergic children to wheeze when exposed to increased EC, NO_2_, or PM_10–2.5_. ORs were largest among the subgroup of boys with mild intermittent asthma for all pollutants except O_3_. A 3.7-μg/m^3^ increase in EC lag 6 was associated with an OR of 1.70 (95% CI, 1.35–2.13) among boys with mild intermittent asthma, a 52.6% increase in effect size relative to the full group [for effects in additional subgroups, see Supplemental Material, Figures 3–5 (doi:10.1289/ehp.0901292)].

By design, children completed only one panel in the first year of the study. To assess whether the greater variability in the wheeze time series in the first year [see Supplemental Material, Figure 1 (doi:10.1289/ehp.0901292)] had an impact on risk estimates, we repeated each of the above analyses with the first year of data removed (9.3% of the panel-days, November 2000 to November 2001). For each of the pollutants, point estimates were similar to the previous findings, although the precision of estimates decreased, presumably because of the loss of 1 year of data (see Supplemental Material, Table 9). We repeated all analyses with the 27 siblings removed (10.3% of panel-days). Point estimates and standard errors changed little (see Supplemental Material, Table 10).

For the full group, when NO_2_ and PM_10–2.5_ were included in the same model, the effect estimates for the association between pollutant and wheeze were reduced by 39% and 10%, respectively, relative to effects in single-pollutant models, and the 95% CIs for both pollutants included 1.00. In the two-pollutant models for the atopy subgroups, only slight changes in magnitude and precision of effect estimates were observed. The magnitude of associations for both NO_2_ and PM_10–2.5_ and wheeze decreased in the subgroup of boys with mild intermittent asthma, and only the association for NO_2_ remained statistically significant [see Supplemental Material, Table 11 (doi:10.1289/ehp.0901292)].

When PM_10–2.5_ and PM_2.5_ were included in the same model, both pollutants were significantly associated with wheeze in the subgroup of boys with mild intermittent asthma, with point estimates that were similar to those observed in single-pollutant models.

## Discussion

We hypothesized that asthmatic children who were sensitized to environmental allergens would be more susceptible to daily changes in ambient air pollutants. We found that increased daily levels of NO_2_ and PM_10–2.5_ were independently associated with increased daily risk of wheeze in models in which both pollutants were included. Children who were skin-test positive to cat or common fungi (*Alternaria* and *Cladosporium*) were more responsive to these pollutants. EC and NO_3_ also were associated with wheeze in one or both of these specifically sensitized subgroups in single-pollutant models. However, children who were skin-test positive to other antigens, including indoor allergens such as house dust mite and cockroach, did not show increased responses to these air pollutants. In a post hoc comparison, the group of boys with mild intermittent asthma had increased risk of wheeze with exposures to NO_2_, PM_2.5_, EC, NO_3_, and PM_10–2.5_ relative to the full group of subjects. O_3_ was not significantly associated with wheeze either in the full cohort or in subgroup analyses.

To our knowledge, this is the first study to show an association of the coarse fraction of PM (PM_10–2.5_) and daily wheeze in asthmatic children. The coarse fraction results may be attributable to coincidence of seasonal trends in concentration with fungal spores (Hjelmroos-Koski et al. 2006). In samples of total suspended particulate (TSP) taken in three locations in California’s South Coast Air Basin, 5–12% of the allergenicity of TSP was attributable to paved road dust (Miguel et al. 1999). The road dust contained allergens from *Cladosporium*, *Alternaria*, and cat (in addition to outdoor pollens) and other materials such as brake and tire fragments that might react with these allergens. Although many *Alternaria* and *Cladosporium* spores are larger, a substantial proportion can be of coarse-fraction size (Misaghi et al. 1978; Rantio-Lehtimaki 1989).

Exposures to fungal antigens may be a major contributor to wheeze in children with asthma. In a panel study of such children, Delfino et al. (1996) showed that daily concentrations of *Alternaria* and *Cladosporium* were associated with increased asthma symptoms, with the associations being largest for those with specific sensitization to fungal antigens. Ostro et al. (2001) also showed that daily concentrations of *Alternaria* and *Cladosporium* were associated with increased risk of wheeze among asthmatic children. More recently, Atkinson et al. (2006) observed that exposure to fungal spores was associated with exacerbations of asthma in London, independent of associations with pollen counts and daily concentrations of black smoke, SO_2_, O_3_, and NO_2_.

Other studies that have evaluated associations between coarse PM and asthma have provided mixed results. Two studies (Lin et al. 2002; Tecer et al. 2008) compared the effects of PM_10–2.5_, PM_2.5_, and PM_10_ on hospitalizations of children for asthma. Lin et al. (2002) found an effect for PM_10–2.5_ but not for PM_2.5_, after adjustment for daily weather conditions. Tecer et al. (2008) found that PM_2.5_ had larger effects than did PM_10–2.5_; however, both were significantly associated with hospitalizations for asthma after adjustment for daily weather conditions. In a study of African-American children with asthma in the Los Angeles area, Ostro et al. (2001) observed that PM_10_ had a greater effect than PM_2.5_ on daily wheeze, although analyses for PM_10–2.5_ were not provided. Two French studies found no association between wheeze episodes or prevalence and PM < 13 μm in aerodynamic diameter or black smoke in either the wintertime or warm seasons among asthmatic children residing in Paris (Just et al. 2002; Segala et al. 1998). An association of either asthma outpatient admissions or beta-agonist prescriptions with PM_10_, but no such association with PM_2.5_, was observed among children in two-pollutant models in Anchorage, Alaska (USA); however, PM_10–2.5_ concentrations were not evaluated (Chimonas and Gessner 2007).

We also observed some effect of PM_2.5_ on daily wheeze. This effect may have been due to the presence of crustal material in PM_2.5_ because such material can constitute up to 20% of fine particulate in the Fresno area (Chow 1995). Alternatively, the method that we used to measure PM_10–2.5_ could have resulted in values that included some fine particles. Although it is possible that fine-particle contamination of PM_10–2.5_ measurements could have accounted for the associations seen, this seems unlikely, given the independent associations we found between PM_10–2.5_ and wheeze in two-pollutant models that included PM_2.5_.

Associations that we observed between NO_2_ and daily wheeze were independent of associations with PM_10–2.5_ and were most pronounced in the group of boys with mild intermittent asthma. The associations with NO_2_ were 12–15% greater in the subgroups that were sensitized to fungal and cat antigens, compared with all subjects. In the Fresno/Clovis study area, the main source for NO_2_ is traffic; therefore, NO_2_ is a reasonable marker for traffic-related pollution (Watson et al. 2000). We have shown previously in this cohort that increased exposure to traffic was related to reduced pulmonary function, with greater effects among those with smaller airways (Margolis et al. 2009). Several other studies have shown that NO_2_ can adversely affect the health of asthmatic children (e.g., Gauderman et al. 2005; Weinmayr et al. 2010). In addition, several controlled human exposure studies have shown that exposure to NO_2_ enhances the bronchoconstrictor responses to inhaled aeroallergen in specifically sensitized adults with asthma (U.S. EPA 2008).

NO_3_ has not typically been evaluated in studies of the effect of air pollutants on asthma. Interest in the respiratory effects of NO_3_ was spurred by the report of Gauderman et al. (2004) on the effect of various air pollutants on growth of lung function in children. Exposure to atmospheric acidity, primarily nitric acid vapor, was associated with decreased growth of lung function in that study. In our analysis, NO_3_ was associated with increased wheeze in the subgroup that was allergic to cat as well as in boys with mild intermittent asthma. The NO_3_ particles that we measured were primarily ammonium nitrate, which is a reaction product of nitric acid vapor and ammonia. Consistent with our finding, Ostro et al. (2009) observed that NO_3_ was associated with hospital admissions for asthma.

Our post hoc observation that boys with mild intermittent asthma were the most responsive to increases in NO_2_, PM_10–2.5_, PM_2.5_, EC, and NO_3_ could be a chance finding. Some investigators have observed greater severity of symptoms in boys (Chimonas and Gessner 2007; Delfino et al. 2004), but the results have been mixed (Lin et al. 2002).

This study does have several limitations. First, because we studied the effects of several pollutants using different time lags in several subgroups of asthmatic children, multiple comparisons were made. However, the associations we found for NO_2_ and wheeze, and to some extent for coarse PM, were relatively stable across multiple time lags and moving averages, which does suggest that these are not due to chance alone. A second limitation may be potential exposure misclassification. We used air quality monitoring data from a central site in Fresno to assign exposures to the various pollutants studied, and we recognize that this may not always accurately represent the actual exposures of our participants. Finally, the results of our study in a relatively small sample of asthmatic children in Fresno may not be generalizable to other populations. Fresno has relatively high levels of both fine and coarse particles compared with other areas of the United States. In addition, the composition of the fine PM in the San Joaquin Valley is considerably different from that of the eastern United States, with more ammonium nitrate and less sulfate.

## Conclusions

We found consistent associations between daily concentrations of PM_10–2.5_ and daily wheeze that were independent of similar associations with NO_2_ and PM_2.5_. These associations were largest in the children who were skin-test positive to fungal and/or cat antigens, which supports our *a priori* hypothesis that asthmatic children with atopy would be most responsive to daily changes in ambient air pollution. Our data suggest the need to identify the components of coarse PM that contribute to asthma morbidity and that particular attention should be paid to the potential importance of the bioaerosol components.

## Figures and Tables

**Table 1:** Selected characteristics of participants at baseline (%).

	Eligible (*n* = 280)	Excluded (*n* = 35)	Full cohort (*n* = 315)
Age (mean ± SD)	8.1 ± 1.7	7.8 ± 1.5	8.1 ± 1.7
Male	57.1	51.4	56.5
Income < $15,000	20.0	20.6	20.4
Home ownership	55.6	62.9	56.5
Health insurance	95.7	97.1	95.0
Hispanic	40.0	37.1	39.7
Non-Hispanic white	41.1	48.6	41.9
African American	15.7	14.3	15.6
Skin-test positive^a^	63.3	57.1	62.7
Mild intermittent asthma^b^	28.2	28.6	28.3
Mild persistent asthma^b^	46.8	54.3	47.6
Moderate or severe asthma^b^	25.0	17.1	24.1
Use inhaled steroids	74.3	62.9	73.0
Oral prednisone, preceding 12 months	38.0	34.3	37.5
FEV_1_ < 80% predicted^c^	17.1	16.1	17.5
FEF_25–75_ < 70% predicted^c^	25.3	32.3	26.2

Abbreviations: FEF_25–75_, forced expiratory flow between 25% and 75% of vital capacity; FEV_1_, forced expiratory volume in 1 sec.

a Positive to at least one allergen on skin-test panel or reported history of severe reaction to prior allergy skin test.

b Based on Global Initiative for Asthma symptom severity guidelines [see Supplemental Material, Table 1 (doi:10.1289/ehp.0901292)].

c Prebronchodilator value.

**Table 2:** Distribution of pollutants and weather conditions monitored at the central site, Fresno, California, from November 2000 to March 2005.

Pollutant	Min	25%	50%	75%	Max	IQR	Increase^a^
NO_2_ (ppb)	4.6	12.9	18.6	24.7	52.4	11.8	8.7
PM_2.5_ (μg/m^3^)	2.0	11.5	18.7	32.0	137.0	20.5	36.2
NO_3_ (ppb)	0.2	1.2	2.5	6.2	32.2	5.0	5.6
EC (μg/m^3^)	0.0	0.7	1.3	2.4	16.7	1.7	3.7
PM_10–2.5_ (μg/m^3^)	0.2	8.4	18.5	31.2	121.0	22.8	14.7
O_3_ (8-hr average; ppb)	3.7	27.2	49.4	69.5	120.0	42.3	20.0
Temperature (°C)	3.0	11.3	16.6	24.5	34.8	13.2	NA
Relative humidity (%)	18.8	42.8	58.0	75.9	97.9	33.1	NA

Abbreviations: %, percentile; IQR, interquartile range; Max, maximum; Min, minimum; NA, not applicable. All metrics are 24-hr averages unless otherwise noted.

a Increase is the 90th percentile of absolute differences in lag 0 and lag 1 concentrations in the peak season (NO_2_, PM_2.5_, NO_3_, and EC, October–February; O_3_ and PM_10–2.5_, April–October). This concentration increase was used for calculation of ORs. The IQR, sometimes used in air pollution epidemiology studies for the interval increase, is listed as a point of comparison.

**Table 3:** Association of pollutants at “representative lag” and wheeze (*n* = 15,252 panel-days).

Pollutant	Lag	OR^a^ (95% CI)	Increase^b^
NO_2_ (ppb)	2	1.10 (1.02–1.20)*	8.7
PM_10–2.5_ (μg/m^3^)	3	1.11 (1.01–1.22)*	14.7
NO_3_ (μg/m^3^)	5	1.05 (0.95–1.16)	5.6
EC (μg/m^3^)	6	1.12 (0.97–1.30)	3.7
PM_2.5_ (μg/m^3^)	5	1.09 (0.93–1.27)	36.2
O_3_ (8-hr average; ppb)	1	1.01 (0.92–1.12)	20.0

All metrics are 24-hr averages unless otherwise noted. “Representative lag” is the one associated with the largest coefficient in the first 7 days.

a Models adjusted for fitted daily mean wheeze, home ownership, smoking in the home, white non-Hispanic ethnicity, moderate or severe asthma severity at baseline, male sex, minimum temperature, home visit group, 6-month interval of entry into cohort, and repeated measures.

b Increase used for OR per 90th percentile of the absolute value of daily differences (lag 0– lag 1) in the pollutant’s peak season across the study period.

* *p* < 0.05.

**Table 4:** Association between air pollution at “ representative lag” and wheeze in selected subgroups of the FACES cohort.

Subgroup/pollutant	Lag	OR (95% CI)
Allergy to cat dander (*n* = 49 children, 2,869 panel-days)		
NO_2_ (ppb)	2	1.27 (1.06–1.51)*
PM_10–2.5_ (μg/m^3^)	3	1.28 (1.09–1.51)*
NO_3_ (μg/m^3^)	5	1.21 (1.01–1.45)*
EC (μg/m^3^)	6	1.33 (1.04–1.71)*
PM_2.5_ (μg/m^3^)	5	1.23 (0.94–1.62)
O_3_ (ppb)	1	0.93 (0.73–1.19)

Allergy to fungi (*n* = 85 children, 4,943 panel-days)		
NO_2_ (ppb)	2	1.23 (1.10–1.39)*
PM_10–2.5_ (μg/m^3^)	3	1.16 (1.02–1.33)*
NO_3_ (μg/m^3^)	5	1.12 (0.97–1.29)
EC (μg/m^3^)	6	1.30 (1.06–1.59)*
PM_2.5_ (μg/m^3^)	5	1.16 (0.94–1.44)
O_3_ (ppb)	1	1.06 (0.92–1.23)

Boys with mild asthma (*n* = 47 children, 2,901 panel-days)		
NO_2_ (ppb)	2	1.51 (1.23–1.85)*
PM_10–2.5_ (μg/m^3^)	3	1.35 (1.10–1.65)*
NO_3_ (μg/m^3^)	5	1.25 (1.03–1.52)*
EC (μg/m^3^)	6	1.70 (1.37–2.12)*
PM_2.5_ (μg/m^3^)	5	1.41 (1.12–1.77)*
O_3_ (ppb)	1	0.86 (0.65–1.13)

“Representative lag” is the one associated with the largest coefficient in the first 7 days.

* *p* < 0.05.
